# Regulatory mechanisms of macrophage polarization in adipose tissue

**DOI:** 10.3389/fimmu.2023.1149366

**Published:** 2023-05-22

**Authors:** Dun Pan, Guo Li, Chunlin Jiang, Jinfeng Hu, Xiangming Hu

**Affiliations:** ^1^ Fujian Key Laboratory of Translational Research in Cancer and Neurodegenerative Diseases, School of Basic Medical Sciences, Fujian Medical University, Fuzhou, China; ^2^ Department of Gastrointestinal Surgery, The First Affiliated Hospital of Fujian Medical University, Fuzhou, China

**Keywords:** adipose tissue macrophages, polarization, organokines, signaling pathway, obesity

## Abstract

In adipose tissue, macrophages are the most abundant immune cells with high heterogeneity and plasticity. Depending on environmental cues and molecular mediators, adipose tissue macrophages (ATMs) can be polarized into pro- or anti-inflammatory cells. In the state of obesity, ATMs switch from the M2 polarized state to the M1 state, which contributes to chronic inflammation, thereby promoting the pathogenic progression of obesity and other metabolic diseases. Recent studies show that multiple ATM subpopulations cluster separately from the M1 or M2 polarized state. Various factors are related to ATM polarization, including cytokines, hormones, metabolites and transcription factors. Here, we discuss our current understanding of the potential regulatory mechanisms underlying ATM polarization induced by autocrine and paracrine factors. A better understanding of how ATMs polarize may provide new therapeutic strategies for obesity-related diseases.

## 1 Introduction

Obesity has become a major public health problem because it increases the risk of many diseases (e.g., type 2 diabetes mellitus, hypertension, osteoarthritis and several cancers) (1). Obesity is characterized by accumulation of adipose tissue (AT), which leads to infiltration of immune cells and chronic low-grade inflammation (2, 3). Being a major portion of AT immune cells, adipose tissue macrophages (ATMs) are key for healthy adipose homeostasis but can also contribute to the pathogenic progression of obesity and other metabolic diseases (4). ATMs can be polarized into distinct phenotypes under different physiological or pathological conditions. In addition to the pro-inflammatory M1 phenotype and anti-inflammatory M2 phenotype (5), several novel ATM subpopulations (e.g., MMe, Mox and LAMs) have been discovered in recent years (6–8). Polarization of ATMs are regulated by organokines and related signaling pathways. In this article, we summarize the characteristics and functions of different ATM subpopulations. In addition, we also highlight the regulatory pathways and mechanisms of ATM polarization, providing novel insights for the treatment of obesity-diseases.

## 2 Polarization and function of ATMs

Many studies have classified macrophages according to the M1/M2 system, which is revealed by the secretory profile of cytokines and interleukins (9). Previous studies have described a ‘‘mixed’’ M1/M2 phenotype for ATMs that can be activated into a switching phenotype (10, 11). ATMs are mainly polarized towards the pro-inflammatory M1 phenotype in the obesity state, conversely, in the lean state, ATMs are polarized towards the anti-inflammatory M2 phenotype (5, 12). As technologies have advanced, accumulated evidence has suggested that ATM polarization is more variable than the M1 or M2 state. Distinct ATM subpopulations express specific markers and have unique transcriptional profiles and functions (as summarized in **Table 1**).

**Table 1:** Adipose tissue macrophages (ATMs) subpopulations.

	M1		M2		MMe	Mox	LAM
**Markers**	Human	Mouse	Human	Mouse	Human/mouse	Mouse	Human/mouse
	CD68, CD11c, CD86	F4/80, CD11b, CD11c	CD68, CD206, CD163	F4/80, CD11b, CD301, CD206	ABCA1, CD36, PLIN2	HO-1, Txnrd1, Srnx-1	CD9, CD36, TREM2
**Functions**	Production of reactive oxygen species for bacterial killing		Promotion of preadipocyte survival, tissue healing, resolution of inflammation		Eliminate dead adipocyte debris	Response to oxidized phospholipids (OxPLs) by upregulating Nrf2-dependent antioxidant enzymes	Counteract inflammation and adipocyte hypertrophy
**Secreted factors**	Galectin-3, resistin; IL-1; IL-18; IL-6; TNF-α		IL-10, IL-1RA, TGF-β, Protectin		IL-6, TNF-α, IL-1β (NOX2-dependent)		
**Induced by**	IFN-γ, TLR4, Saturated FFA, Aldosterone, LTB4, Ceramides, Type I interferons, PAMP/DAMP, LPS		Prostaglandin D2, IL-4, Meteorin-like, Adiponectin, IL-10, IL-13, T regulatory cells, Eosinophils, FAHFA, AMPK		High levels of glucose, insulin, and palmitate	Oxidized phospholipids, Nrf2	Trem2
**Metabolic effects**	Promote IR, Decrease UCP1		Promote insulin sensitivity, Increase UCP1, Promote mitochondrial health			Suppression of regular energy metabolism	Preventing adipocyte hypertrophy and loss of systemic lipid homeostasis under obese conditions
**Dominant polarization**	Obese adipose tissue		Lean adipose tissue		Obese adipose tissue	Lean adipose tissue	Obese adipose tissue
**References**	(13, 14)		(13, 14)		(6)	(7)	(12)

### 2.1 Classical activated macrophages (M1)

Obese AT is recognized as a low-grade, chronic inflammation condition accompanied by accumulation of ATMs and a phenotypic switch (2, 3). Obese adipocytes secrete various chemokines and adipokines (e.g., MCP-1, CXCL12 and Leptin), which recruit and switch macrophages from the M2 state to the M1 state (5). ATMs are recognized as cells that co-express F4/80 and CD11b in mice or CD68 and CD11b in human (2, 15). Moreover, CD11c is used as phenotypic markers of M1-ATMs, which produce pro-inflammatory mediators like IL-1β, TNF-α and nitric oxide (NO), acting as main effectors of inflammatory signals, impaired adipocyte function and insulin sensitivity (13, 14). Ablation of CD11c positive cells alleviated inflammation and improved insulin sensitivity in obese mice (16). The significance of M1-ATMs is also supported in human studies where CD11c positive ATMs has been associated with glucose intolerance and metabolic syndrome (15).

### 2.2 Alternatively activated macrophages (M2)

Unlike pro-inflammatory M1-ATMs, M2-ATMs attenuate inflammation to maintain adipose homeostasis (5). In the lean state, the dominant ATMs are considered as resident macrophages which express markers of M2 macrophages (e.g., CD206, CD301 and CD163) (2, 5). M2-ATMs are further divided into three major subtypes: M2a, M2b and M2c (17), which express specific markers and have unique functions. M2a-TAMs are characterized by high surface expression of IL-R and FIZZ1, and secrete TGF-𝛽, IGF and fibronectin to contribute to tissue repair (18, 19). M2b-TAMs express high levels of IL-10, CCL1 and TNFSF14, and low levels of IL-12, exhibiting anti-inflammatory and immune-regulated effects (20). M2c-TAMs expressing multiple markers like CD14 and TLR1, have high expression of IL-10, TGF-𝛽 and Mer receptor tyrosine kinase, and are considered as anti-inflammatory and phagocytic macrophages (17).

### 2.3 Metabolically activated macrophages

ATMs have a particular metabolically activated phenotype called “MMe”, which exhibit a mixture of M1 and M2 characteristics. The MMe phenotype, which can be identified by their surface markers CD36, ABCA1, and PLIN2, is stimulated by high levels of glucose, insulin, and palmitate (6). MMe macrophages not only promote insulin resistance (IR) *via* producing inflammatory cytokines, but also clear away dead adipocyte through lysosomal exocytosis, which protect AT from the deleterious effects of excess free fatty acids (FFAs) (21). Therefore, MMe macrophages perform both detrimental and beneficial functions during obesity.

### 2.4 Oxidized macrophages

Recently, a novel macrophage phenotype has been identified, known as Mox, mainly stimulated by oxidized lipids (7). High expression of Txnrd-1, Srnx-1 and HO-1 distinguishes Mox from the M1 or M2 phenotype. Compared with M1- and M2-TAMs, Mox macrophages exhibit restricted bioenergetics and more antioxidant production. Recent study has shown that Mox macrophages are the predominant ATMs in lean AT, while more energetic macrophages like M1- or M2-TAMs predominate during the development of obesity (7).

### 2.5 Lipid-associated macrophages

Recently, a novel ATM subpopulation defined as lipid-associated macrophages (LAMs) were discovered surrounding apoptotic adipocytes of obese AT (8). LAMs express highly conserved genes, including CD9, CD36, and the lipid receptor Trem2. LAMs utilize Trem2 as an extracellular lipid sensor and perform protective functions to combat adipocyte inflammation, hypertrophy, and metabolic dysfunction (22). In addition, LAMs express many immunosuppression-related genes such as Lgals1/3, suggesting that they may be involved in regulating inflammatory response induced by lipid accumulation (8).

## 3 Organokines: Integrators of ATM polarization

Accumulating evidences suggest that AT, liver, skeletal muscle and gut function as endocrine organs, producing various organokines (adipokines, hepatokines, myokines and gut cytokines) that are capable of recruiting macrophages or switching ATM phenotypes.

### 3.1 Adipokines

#### 3.1.1 Pro-inflammatory adipokines

##### 3.1.1.1 Leptin

AT secretes a variety of hormones/cytokines, which are called adipokines. Leptin, the first classical adipokine, is initially considered as a satiety signal that regulates body weight by reducing food intake and increasing energy expenditure (23, 24). Mice with leptin deficiency (ob/ob) or leptin receptor deficiency (db/db) develop morbid obesity due to hyperphagia (23, 25). However, due to leptin resistance, most forms of obesity of animals and human are associated with higher leptin levels rather than leptin deficiency (26).

In addition to its role in energy balance, leptin also functions as an immunomodulatory cytokine, inducing immunologic alterations in different cell types, including ATMs. The immunoregulatory effects of leptin were first assessed in ob/ob mice. Macrophages from ob/ob mice have impaired phagocytic activity and pro-inflammatory cytokine production, and exogenous leptin treatment improves the above defects (27). Leptin treatment in ob/ob mice also ameliorates IR while up-regulating the expression of M2 markers (e.g., Fizz-1, Arg-1 and Mgl-1) (28). Similarly, in vitro leptin treatment, CD14 human macrophages up-regulates typical M2 markers, while being able to increase the expression of M1 markers (e.g., IL-6, IL-1𝛽, and MCP-1) (29). These studies suggest that leptin could be a contributor to the distinct ATMs phenotype. Besides, leptin induces the expression of vascular cell adhesion molecules, which can increase macrophage infiltration into AT (30). Leptin also stimulates macrophage proliferation in a dose-dependent manner (31), meaning obesity-associated hyperleptinemia can increase the proliferation of ATMs. Hence, increased leptin levels in the obese AT are, in part, responsible for accumulation and activation of ATMs.

##### 3.1.1.2 Resistin

The name ‘resistin’ is coined from the original observation that it induces IR and has been proposed to link obesity and diabetes (32). Resistin is a 12.5-kDa peptide hormone, which is predominantly expressed in white adipocytes in rodents whereas in humans its main source is peripheral blood mononuclear cells (PBMCs) and macrophages (33). In mice, circulating resistin levels are positively correlated to obesity and IR, and resistin-treated mice or resistin-overexpressing transgenic mice exhibit glucose intolerance and IR (34). Conversely, resistin knockout or resistin neutralization with antibodies improves insulin sensitivity in diet-induced obese mice (32). However, in humans, the link between increased resistin levels and obesity/IR remains under debate and needs more epidemiological studies (34).

In obese conditions, there is an increase in adipocytes and ATMs leading to increased resistin expression. Resistin induces the expression of chemokines (e.g., CCL2 and CXCL1) as well as adhesion molecules (e.g., ICAM-1 and VCAM-1) to promote monocytes infiltration into a variety of tissues and organs including AT (35). M0 macrophages originated from monocytes can further differentiate into distinct ATM subsets, including LAMs (8), M1 and M2 macrophages (36), under varying circumstances. Resistin originated from adipose resident macrophages stimulates the expression of pro-inflammatory cytokines like IL-6, IL-12 and TNF-α (37), indicating that resistin might promote an M1-like phenotype in ATMs. Thus, resistin works by autocrine, paracrine and endocrine modes, and affects the accumulation and polarization of macrophages in AT.

##### 3.1.1.3 WISP1

Wingless‐type (Wnt)-inducible signaling pathway protein-1 (WISP1), a matricellular protein, is a novel adipokine associated with inflammation in obesity (38–40). WISP1 levels in plasma and subcutaneous AT are elevated in obese subjects and are positively correlated with systemic inflammation and IR (38). WISP1 plays a pro‐inflammatory role in AT inflammation. Stimulation of macrophages with WISP1 induces the secretion of pro-inflammatory cytokines (e.g., TNF-a and IL-6) and promotes M1 macrophage polarization (38). However, in adipocytes, WISP1 neither induces cytokine expression nor affects insulin signaling (38). This suggests that WISP1 contributes to AT inflammation and IR by regulating macrophages rather than adipocytes.

#### 3.1.2 Anti-inflammatory adipokines

##### 3.1.2.1 Adiponectin

As the best-known and most abundant adipokine in circulation, adiponectin is exclusively secreted from adipocytes (41). Unlike pro-inflammatory adipokines, which have a positive trend in conditions of obesity, adiponectin levels are reduced in obesity and are up-regulated after weight loss (42, 43). Many evidences proved that adiponectin shows protective activity in obesity and IR (44, 45).

Adiponectin acts as anti-inflammatory factor and plays important roles in the accumulation and polarization of ATMs. Adiponectin has been reported to promote calreticulin receptor-dependent clearance of apoptotic adipocytes (46). Since apoptosis of adipocytes is a key initial event that contributes to macrophage infiltration into AT (47), the inhibition of adipocyte apoptosis using adiponectin can suppress the infiltration of ATMs. Furthermore, adiponectin induces anti-inflammatory M2 macrophage proliferation in AT by the activation of AKT signaling (48), while suppresses pro-inflammatory M1 macrophage proliferation via inhibiting NF-κB signaling (49). Additionally, adiponectin induces the M1 to M2 macrophage polarization switch in AT. Macrophages from adiponectin deficient mice display increased M1 markers and decreased M2 markers, while systemic administration of adenovirus expressing adiponectin results in an increased expression of M2 markers in AT (50). Overall, adiponectin regulates the infiltration, proliferation, and polarization of ATMs, which accounts for its anti-inflammatory properties.

### 3.2 Hepatokines

#### 3.2.1 Fetuin-A

Fetuin-A is a fatty acid-binding glycoprotein that is primarily expressed in the liver (51). Circulating Fetuin-A levels are higher in human subjects with obesity, type 2 diabetes mellitus (T2DM) and nonalcoholic fatty liver disease (NAFLD) (52). In obese AT, fatty acid-binding Fetuin-A acts as an endogenous ligand of TLR4 to promote inflammation and IR (53). By binding to the extracellular domain of TLR4, Fetuin-A activates NF-κB signaling to induce pro-inflammatory cytokines release and promote M1 macrophages polarization (53, 54). Moreover, Fetuin-A also serves as a chemoattractant, inducing the infiltration of macrophages into AT (55).

#### 3.2.2 GDF-15

Growth differentiation factor 15 (GDF-15), a distant member of the TGF-β superfamily, is highly expressed in the liver (56) and AT (57). GDF-15 has been shown to be a stress responsive cytokine associated with obesity and diabetes (58). GDF-15 exerts its known anti-inflammatory properties against obesity by regulating at least in part the activation of ATMs. GDF-15 transgenic mice fed a HFD exhibit reduced NLRP3 inflammasome activity and lower levels of macrophage infiltration into AT (59).Recently, a study suggests that GDF-15 plays a role in the polarization of ATMs. GDF-15 expression in macrophages is induced by IL-4, which promotes M2 polarization of ATMs via the upregulation of oxidative metabolism (60).

### 3.3 Myokines

#### 3.3.1 Irisin/FNDC5

Irisin, a 12 kDa peptide, is a novel myokine that is cleaved from fibronectin type III domain protein 5 (FNDC5) (61). In obese subjects, circulating irisin levels are reduced and are related with insulin sensitivity (62). Irisin has been reported to ameliorate adipose inflammation *via* up-regulating anti-inflammatory cytokine (e.g., adiponectin) and down-regulating pro-inflammatory cytokine (e.g., leptin and IL-6) (63). FNDC5 overexpression attenuates adipose tissue inflammation in HFD-induced obese mice by inhibiting macrophage recruitment and M1 phenotype polarization (64).Therefore, irisin’s anti-inflammatory effect in AT includes reducing production of pro-inflammatory cytokines, suppressing macrophage proliferation and infiltration, and inducing M2 macrophage polarization.

#### 3.3.2 Myostatin

Myostatin (MSTN), also termed as growth differentiation factor 8, is primarily produced by skeletal muscle, and negatively regulates skeletal muscle mass. A recent study has reported that serum myostatin levels are positively correlated with adipose inflammation, obesity and IR (65). Inhibition of myostatin in mice suppresses HFD-induced infiltration of macrophages and reduces the expression of pro-inflammatory cytokines in AT (66). In addition, myostatin inhibition increases irisin production and induces M2 macrophage polarization in AT, thus suppressing inflammation (66).

### 3.4 Gut cytokines

#### 3.4.1 GLP-1

GLP-1 is a hormone secreted by intestinal L-cells and is associated with obesity-related inflammation. GLP-1 alleviates macrophage infiltration in AT of ob/ob mice and reduces M1-polarized specific mRNA expression (67). Another study has shown that GLP-1/GLP-1R signaling in macrophages suppresses M1 polarization and triggers M2 polarization (68).In light of these findings, we speculate that GLP-1 alleviates obesity-related inflammation *via* inhibiting macrophage recruitment and promoting M2 macrophage polarization in AT.

#### 3.4.2 Ghrelin

Ghrelin is a gastrointestinal cytokine that increases appetite and promotes obesity (69). A number of studies have described that ghrelin has strong anti-inflammatory properties, however, the effects of ghrelin in macrophages are complex. *In vitro* model, ghrelin inhibits LPS-induced production of pro-inflammatory cytokines in macrophages (70). Ghrelin’s function is mediated by its receptors GHSR. *In vivo* work has demonstrated that GHSR knockout or administration of Des-acyl ghrelin (functions as ghrelin antagonist), in obese mice, reduces macrophage infiltration, promotes macrophage polarization to M2 in AT, thus suppressing adipose inflammation (71, 72). These data suggest that Ghrelin/GHSR axis can act as a pro-inflammatory mediator in AT.

#### 3.4.3 Other organokines

**Figure 1:**
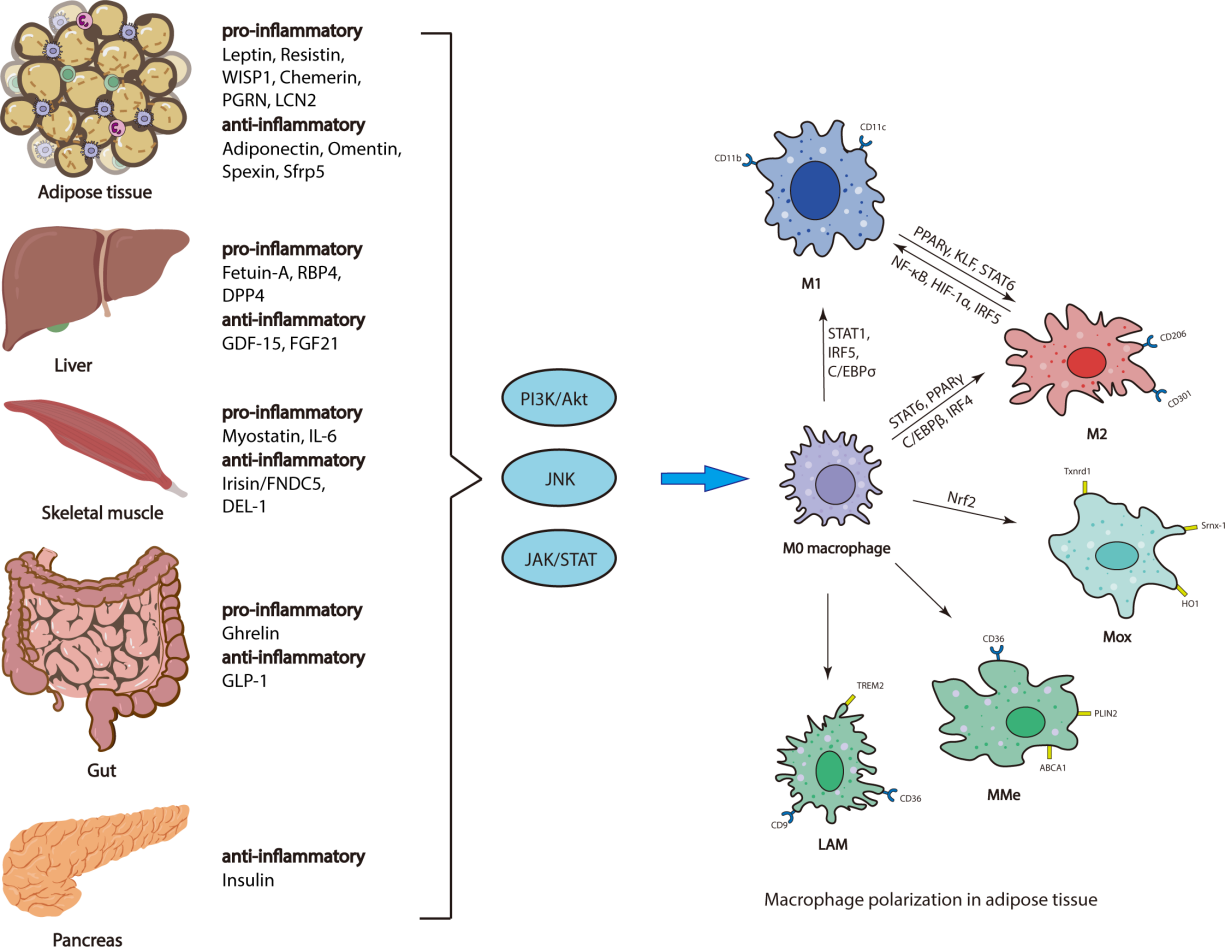
Regulatory mechanisms of ATM polarization. Adipose tissue, liver, skeletal muscle, gut and pancreas secrete various organokines, which are increased or decreased, initiating multiple downstream signaling pathways, thus affecting ATM polarization.

Studies have demonstrated that several other organokines, including adipokines (pro-inflammatory: Chemerin, PGRN and LCN2; anti-inflammatory: Omentin, Spexin, and Sfrp5), Hepatokines (pro-inflammatory: RBP4 and DPP4; anti-inflammatory: FGF21), Myokines (pro-inflammatory: IL-6; anti-inflammatory: DEL-1) and insulin are important players in regulation of the recruitment or polarization of macrophages in AT, and thus promote inflammation associated with obesity and metabolic disease (73, 74) (**Figure 1**).

## 4 Regulatory pathways of ATM polarization

The past decades have revealed several key regulatory pathways of ATM polarization.

### 4.1 PI3K/Akt signaling pathway

The PI3K singling cascade is a central metabolic regulator, which is activated by metabolic stimuli including insulin, glucose, FFAs and various organokines. ATMs are exposed to increased levels of these stimuli in the obese state and adopts metabolism-dependent phenotypes. Numerous studies have implicated that PI3K/AKT singling plays an inhibitory role in TLRs-mediated inflammation and contributes to M2 macrophage polarization (75). Obesity is associated with increased circulating LPS, which initiates adipose inflammation and macrophage activation by activating TLR4 (76), suggesting PI3K/AKT singling can regulate ATM polarization. Indeed, a recent study has demonstrated that macrophage-intrinsic PI3K signaling promotes a beneficial ATM population characterized by lipid uptake (77). It is worth noting that Akt isoforms differentially contributes to macrophage polarization, with AKT1 ablation induing M1 activation and AKT2 ablation resulting in M2 phenotype activation (78). However, how individual Akt isoforms are activated by PI3Ks in the context of macrophages polarization remains to be elucidated.

### 4.2 C-Jun N-terminal kinase signaling pathway

Mixed-lineage kinase 3 (MLK3) deficiency attenuates JNK activation, reduces ATM accumulation and M1 activation in HFD-fed mice (79). In macrophages, PPARγ is efficiently phosphorylated by JNK (80). PPARγ knockout in murine myeloid cells induces M1 activation of ATMs, obesity and IR (81). Acute exercise improves insulin signaling in the White AT, at least in part by inducing macrophage polarization to M2 *via* down-regulating phospho-JNK (82). These studies have demonstrated that JNK signaling plays a pivotal role in both ATM accumulation and polarization.

### 4.3 JAK/STAT signaling pathway

IFNγ is the best known inducer of JAK-STAT signaling (83). In recent studies, deletion of either TRIM29 or TRIM18 increases the release of IFNγ that enhances inflammatory cytokine production and induces M1 macrophage polarization through activating STAT1 (84–86). SOCS proteins act as feedback inhibitors of the JAK/STAT signaling. Myeloid SOCS3 knockout mice exhibit prolonged activation of JAK/STAT signaling and increased expression of pro-inflammatory cytokines in macrophages (87). In contrast to STAT1, STAT6 promotes M2 macrophage polarization. Activation of STAT6 by IL-4 attenuates adipose inflammation by inducing proliferation of local ATMs and polarization of M2 phenotype (88).

## 5 Conclusion

The infiltration and polarization of macrophages in adipose tissue are beginning to be recognized as pivotal instigators of metabolic dysfunction and obesity. In response to over-nutrition, endocrine organs change metabolic phenotypes, release distinct secretome profiles, and shift adipose homeostasis into one that promotes macrophage invasion and polarization, and supports downstream chronic inflammation and IR.

Several endocrine organs are involved in regulation of ATM polarization, including adipose tissue, gut, liver, skeletal muscle and pancreas. Various organokines derived from these endocrine organs bind to their respective receptors to initiate multiple downstream signaling pathways (e.g., PI3K/AKT, JNK, and JAK/STAT), which regulate ATM polarization *via* different transcriptional regulators (e.g., PPARγ, STAT, C/EBP and IRFs) (**Figure 1**).

It’s worth noting that the increase in the M1/M2 ratio of ATMs during obesity cannot be explained simply by the transformation from M2- to M1-phenotype macrophages but rather by the infiltration of circulating monocytes to AT followed by differentiating into M1 and M2 macrophages (36, 89). Mouse ATMs can be generated from circulating monocytes classified as Ly6C^+^ and Ly6C^−^ that are generally thought to preferentially differentiate into M1 and M2 macrophages, respectively (17). Human CD14^+^ CD16^−^ and CD14^+^ CD16^+^ monocytes are considered to resemble mouse Ly6C^+^ inflammatory monocytes, while CD14^dim^ CD16^+^ monocytes resemble Ly6C^−^ anti‐inflammatory monocytes (17, 90). In human obese AT, pro-inflammatory macrophages have been described as CD14^+^CD16^+^ cells with high levels of M1 markers (91). Polarization of inflammatory monocytes has been implicated in the pathogenesis of obesity-related diseases including T2DM and atherosclerosis (92, 93), thus underlying mechanisms and approaches for resolving monocyte polarization conducive to disease regression need to be established.

Despite the great achievements made over the past few decades, a lot of questions on regulatory mechanisms of ATM polarization and its physiological and pathological functions are yet to be answered. Another limitation in the field is that current ATM-markers are ubiquitously expressed in macrophages in different tissues but not specifically in ATMs. More specific markers need to be identified, which will greatly facilitate our understanding on ATMs in health and metabolic diseases. Our understanding of the complexity of ATM subpopulations is inadequate since macrophages are highly plastic and heterogeneous cell populations. New technologies, including single-cell analysis, computational biology and bioinformatics, are being incorporated in this field and are expected to hopefully help address those challenges. With increasing understanding of regulatory mechanisms of ATM polarization, novel insights and treatment strategies should emerge in the prevention of obesity-related diseases.

## Author contributions

DP, GL and CJ drafted the original manuscript. DP and GL designed and created the figures and tables. JH and XH structured and revised the manuscript. All authors contributed to the article and approved the submitted version.
